# Spectrum of Etiologies Causing Hydrometrocolpos

**Published:** 2013-01-01

**Authors:** Aysenur Cerrah Celayir, Gökmen Kurt, Ceyhan Sahin, Inanç Cici

**Affiliations:** Department of Pediatric Surgery, Zeynep Kamil Maternity and Children's Training and Research Hospital Istanbul, Turkey.

**Keywords:** Hydrometrocolpos, Neonate, Urinary obstruction, Etiology

## Abstract

Background: Hydrometrocolpos (HMC) develops as a result of vaginal outflow obstruction and the accumulation of secretions. It might be secondary to persistent cloaca, urogenital sinus, some syndromes, presence of the vaginal septum, vaginal atresia, and imperforate hymen. Each of them has different treatment options and follow-up protocols. This study was performed to identify the etiology and the related management of patients with HMC.

Materials and Methods: A descriptive series of patients with HMC managed in our hospital between 2004 and 2011 is being presented. The medical record of these patients was analyzed for etiology, management, and outcome.

Results: Eight patients with HMC were managed during 7 years at our department. Underlying etiologies included urogenital sinus (n=3), and 1 each of imperforate hymen, transverse vaginal septum, Herlyn-Werner-Wunderlich syndrome, persistent cloaca, and a variant of the cloaca. Four patients were prenatally diagnosed. The patient with imperforate hymen was managed successfully with incision and drainage. Abdominal vaginostomy was done in three patients with urogenital sinus as initial procedure. In patient with persistent cloaca, a colostomy and abdominal vaginostomy were performed. Patient with cloaca variant died due to persistent acidosis and salt wasting.

Conclusion: HMC may have different etiological factors which may dictate different surgical management. Etiology of HMC can be as simple as imperforate hymen to the most severe cloacal malformations.

## INTRODUCTION

Hydrometrocolpos (HMC) develops in the female as a result of a vaginal outflow obstruction and the accumulation of secretions. It might be secondary to a transverse vaginal septum, vaginal atresia or imperforate hymen, and cloacal anomaly [1-3]. Many of these females may present with associated malformations and syndromes, such as cloacal dysgenesis sequence, McKusick–Kauf-man, Ellis–van Creveld or Bardet–Biedl syndromes [4-6]. The underlying etiology of HMC dictates the surgical management. We describe our experience of 8 cases of HMC with different pathologies leading to variable surgical therapy.

## MATERIALS AND METHODS

The medical record of 8 patients with HMC, managed in our hospital between 2004 and 2011, was analyzed for etiology, management, and outcome.

## RESULTS

Eight patients with a diagnosis of HMC presented to us at different ages (Table 1). Four of them presented for the management of HMC (prenatally diagnosed). Two patients presented with a pelvic mass, whereas, two other patients presented for ambiguous genitalia/anorectal malformation; and HMC was diagnosed while evaluation.

In the subset that was prenatally diagnosed to have HMC, one patient had imperforate hymen leading to the pathology. Two patients had urogenital sinus and one patient had persistent cloaca as etiology of HMC. One of the patients with urogenital sinus also had type IV sacrococcygeal teratoma (SCT). Of those who presented with pelvis mass, one patient was operated for acute abdomen else-where and then referred to us for the management of HMC. On examination, transverse vaginal septum was found. The other patient presented with a mass behind urinary bladder and left renal agenesis. On evaluation, she was found to have bifid uterus and vaginal septum. However, spontaneous drainage of the mass occurred following menstruation and Herlyn-Werner-Wunderlich (HWW) syndrome was assumed as her diagnosis. 

One patient of ambiguous genitalia with HMC had persistent cloaca. The patient however succumbed to persistent metabolic acidosis and electrolyte imbalance, the cause of which could not be identified. In all cases, hydronephrosis secondary to lower urinary tract obstruction was noted. In fetal HMC cases, one had severe oligohydramnios. In one case of urogenital sinus, urinary ascites was noted in prenatal period. The management in these patients is tabulated in table 1.

**Figure:**
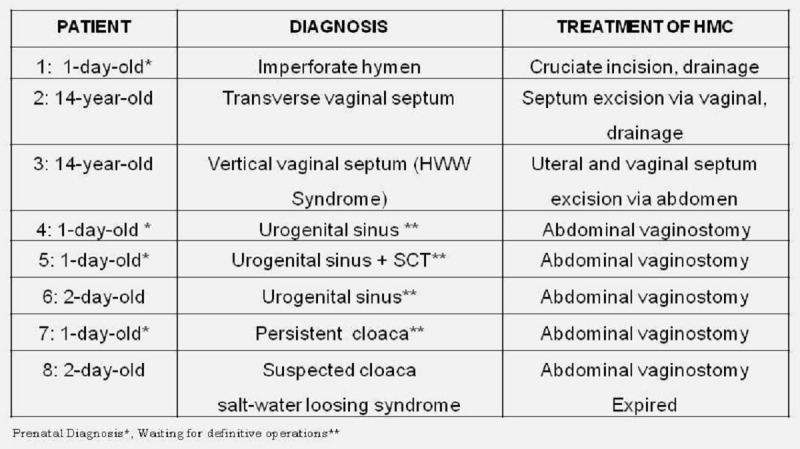
Table 1: Demonstrative properties of the hydrocolpos cases are summarized.

## DISCUSSION

HMC is a manifestation of many different non-syndromic/syndromic anomalies. Imperforate hymen, vaginal atresia, persistent urogenital sinus, and cloacal malformation, are the main etiological factors [1-3]. 

It may account for 15% of lower abdominal masses in females [1-3]. The differential diagnoses of HMC include meconium pseudocyst, enteric duplication cyst, fetal ovarian cyst, dilated bowel, mesenteric cyst, rectal duplication, cystic neuroblastoma, or bladder duplication [4-6].

HMC may be associated with urinary and intestinal tract malformations through a wide spectrum of more complex malformations, from persistent urogenital sinus to cloacal dysgenesis [2-9]. Although these malformations share common embryological origins, they might lead to different anatomical defects [10]. In persistent urogenital sinus, failure of urethrovaginal septation at 6 weeks' gestation leads to drainage of the bladder and the vagina into a common channel. In this case, the perineum exhibits two distinctive orifices, a ventral urogenital sinus and a dorsal anus. In cloacal malformation, complete developmental failure of the urorectal septum, which normally divides the cloaca into a ventral urogenital sinus and a posterior anorectal canal at 5 weeks' gestation, leads to a single exit chamber and severe anorectal atresia. This chamber is connected to a single perineal orifice [10, 11]. An increase in cholinergic innervation in both the cloaca and colorectum during development had been determined in another study [12-15].

Presentation of HMC depends on the degree of compression of the surrounding structures by the uterovaginal swelling [16,17], Commonly, the urinary tract is compressed leading to varying degrees of hydronephrosis [16-19]. Pressure on the bowel to cause obstruction is not common [16,17]. The diagnosis of HMC is often delayed in older girls which often have prolonged investigations to exclude more common causes of intestinal and urinary obstruction [16-18]. HMC should be considered in the differential diagnosis of a female infant with abdominal mass with or without constipation and urinary obstruction.

When hydrocolpos is suspected prenatally, subsequent ultrasound examination should rule out associated malformations and provide precise information of the pelvic anatomy. The contribution of MRI has been excellent for diagnosis when ultrasonography was unable to specify fetal pelvic anatomy [2,4-6,8,9]. Ultrasound and MRI are currently recognized as a valuable tool for imaging. The characteristic features of HMC are as follows: (1) an abdominal mass locate in the midline posterior to the bladder, (2) the mass is fluid-filled with a mid-plane septum, (3) the mass is connected to the dilated uterus, and(4) if a four-chamber appearance is observed, then it is consisting of the septate vagina and two uterine cavities [2,4,6-8]. 

Surgical intervention for isolated HMC solely with lower tract obstruction is associated with excellent results, however, surgery for persistent urogenital sinus is uncertain and even more so for cloacal malformation. Some authors have suggested that in persistent urogenital sinus or cloacal malformation, fetal urine may escape from the fallopian tubes into the abdomen [2,4,5,8,17]. In the 8th case that we have described, biochemical analysis of the ascitic fluid not only confirmed the presence of urine but also demonstrated the presence of digestive enzymes. This information, together with the absence of visualization of the anal canal and rectum, made the diagnosis of cloacal malformation even more likely.

The prenatal differentiation of female urogenital anomalies can be difficult because of their rarity, variations in presentation and poor imaging by ultrasonography, especially in late gestation. Although a prenatal diagnosis of HMC has been reported as early as 25 weeks of gestation, most cases are described in the third trimester of pregnancy or after birth [2,4,6,7,8]. Although the cystic mass could be distinguished from the bladder, which was identified by the surrounding umbilical arteries, the origin of this mass could not be determined by ultrasonography. MRI was useful for identifying the uterus and the rectum using T1-weighted imaging, particularly as it revealed the continuity of the mass with other organs [2,4,6-8].

## CONCLUSION

In conclusion, etiology of HMC is as simple as imperforate hymen to the most severe cloacal malformations. Treatment planning is tailored according to causes of HMC. Parents should be counseled about treatment and prognosis as to the underlying pathology causing HMC.

## Footnotes

**Source of Support:** Nil

**Conflict of Interest:** None
